# Current Status and Future Perspectives of Robotic‐Assisted Redo Pyeloplasty for Recurrent Ureteropelvic Junction Obstruction

**DOI:** 10.1111/iju.70233

**Published:** 2025-09-16

**Authors:** Daisuke Ishii, Kohei Mori, Izuru Shiba, Yutaka Shiono, Kazumasa Matsumoto

**Affiliations:** ^1^ Department of Urology Kitasato University School of Medicine Sagamihara Kanagawa Japan

**Keywords:** recurrent ureteropelvic junction obstruction, redo pyeloplasty, robotic surgery, ureteropelvic junction obstruction

## Abstract

Recurrent ureteropelvic junction obstruction presents significant surgical challenges due to fibrosis, altered anatomy, and impaired vascularity. Open redo pyeloplasty has traditionally been the gold standard; however, it is associated with considerable morbidity. Minimally invasive options such as endopyelotomy and laparoscopic pyeloplasty offer faster recovery but have lower success rates and greater technical complexity. In this review, we aimed to provide a comprehensive evaluation of the current literature on robot‐assisted surgical management of recurrent ureteropelvic junction obstruction. Robot‐assisted laparoscopic pyeloplasty has emerged as a promising alternative, providing enhanced visualization, improved dexterity, and precise suturing, even in reoperative cases. Redo robot‐assisted laparoscopic pyeloplasty achieves high success rates (75%–100%) with low complication rates and shorter hospital stays compared with open or conventional laparoscopic approaches. Moreover, advanced robotic techniques—including ureterocalicostomy, buccal mucosa graft ureteroplasty, ileal ureter replacement, and renal autotransplantation—have broadened reconstructive options for complex or recurrent cases. Single‐port robotic platforms are also gaining interest for their potential to reduce invasiveness and improve cosmetic outcomes. Despite favorable outcomes, existing evidence is primarily limited to retrospective, single‐institution studies. Further prospective, multicenter research is needed to validate long‐term efficacy and cost‐effectiveness. Surgeon expertise, careful patient selection, and adherence to standardized protocols remain essential for optimizing outcomes. As robotic technology continues to evolve, robotic redo pyeloplasty and it's adjunct procedures are increasingly considered preferred treatment modalities for recurrent or anatomically complex ureteropelvic junction obstruction.

**Abbreviations**
BMG: buccal mucosa graft
EBL: estimated blood loss
ICG: indocyanine green
MAG3: mercaptoacetyltriglycine
RA‐BMG: robot‐assisted buccal mucosa graft ureteroplasty
RA‐IUR: robot‐assisted ileal ureter replacement
RA‐KAT: robot‐assisted kidney autotransplantation
RAP: robot‐assisted pyeloplasty
RAUC: robot‐assisted ureterocalicostomy
SARA: supine anterior retroperitoneal access
SP: single port
SP‐RAP: single‐port robot‐assisted pyeloplasty
SP‐RAUC: single‐port robot‐assisted ureterocalicostomy
TIT: total ischemic time
UPJO: ureteropelvic junction obstruction
WIT: warm ischemic time

## 1 Introduction

Ureteropelvic junction obstruction (UPJO) is one of the most common causes of hydronephrosis in pediatric and adult populations. If left untreated or inadequately managed, UPJO can lead to progressive renal function deterioration and long‐term complications. Primary pyeloplasty, most often performed using the Anderson–Hynes dismembered technique, is widely accepted as the standard treatment, with reported success rates between 90% and 95%. However, postoperative obstruction recurs in approximately 5%–15% of cases, requiring additional surgical intervention in some patients [1, 2]. Managing recurrent UPJO poses significant challenges. Compared with primary procedures, redo surgeries are more complex owing to dense fibrosis, distorted anatomical landmarks, and impaired vascularity, all of which increase complication risk and technical difficulty [3, 4]. Traditionally, open redo pyeloplasty has been considered the gold standard for repeat procedures, providing reliable outcomes but with increased morbidity, longer hospital stays, and delayed recovery [5]. To reduce the invasiveness of reoperations, minimally invasive techniques such as laparoscopic pyeloplasty and endourological interventions—including endopyelotomy—have been introduced. Laparoscopic surgery is less invasive; however, it can be technically challenging in the presence of postoperative adhesions or scarred tissues and requires advanced suturing techniques. Endoscopic approaches often yield low success rates, particularly in cases involving crossing vessels or long stricture segments [6, 7]. More recently, robot‐assisted laparoscopic pyeloplasty (RAP) has emerged as a promising option for managing recurrent UPJO. Robotic systems offer enhanced three‐dimensional visualization, improved instrument articulation, and superior ergonomics, which significantly facilitate complex reconstructive procedures [8]. Initial studies suggest that redo RAP achieves success rates comparable to or greater than those of open and conventional laparoscopic approaches, with additional benefits including reduced complications, shorter hospital stays, and faster postoperative recovery [9, 10]. Furthermore, as robotic platforms have evolved, their application has expanded beyond standard pyeloplasty to include alternative reconstructive techniques such as buccal mucosa graft ureteroplasty, ureterocalicostomy, ileal ureter replacement, and even renal autotransplantation. These approaches are increasingly used to manage recurrent or anatomically complex UPJO cases that are not amenable to standard procedures. However, most evidence supporting redo RAP comes from single‐center retrospective case series and small cohort analyses. Therefore, further validation through multicenter studies with long‐term follow‐up is needed to better define its role in treating recurrent UPJO [11]. In this review, we aimed to provide a comprehensive evaluation of the current literature on robot‐assisted surgical management of recurrent UPJO—including redo pyeloplasty and other advanced reconstructive strategies—highlighting clinical outcomes, technical considerations, and future directions.

## 2 Historical Perspectives on Redo Pyeloplasty

The surgical management of recurrent UPJO has evolved significantly over the past several decades. First described by Anderson and Hynes in 1949, open pyeloplasty became the gold standard because of its high success rates and effective anatomical reconstruction [12]. However, despite favorable outcomes, recurrent obstruction occurred in approximately 5%–15% of cases, requiring redo surgery [4].

Traditionally, recurrent UPJO was managed with open pyeloplasty, providing robust success but at the cost of increased patient morbidity, prolonged hospitalization, and delayed recovery [1, 4]. The introduction of minimally invasive techniques in the late 20th century transformed the management of recurrent UPJO. Endopyelotomy, introduced in the 1980s, gained popularity as a less invasive option. It is performed via retrograde ureteroscopy or percutaneous antegrade access and offers shorter operative times, reduced postoperative pain, and faster recovery [13]. However, its success rates—typically 60%–75%—are significantly lower than those of redo open surgery, particularly in cases with dense fibrosis or extrinsic compression from crossing vessels [6, 13].

The first successful laparoscopic reconstruction of UPJO was performed by Schuessler in 1993 [14]. Since then, laparoscopic surgery for recurrent UPJO has also been reported [6, 7, 15, 16]. However, it remains technically challenging owing to factors such as fibrosis, adhesions, altered anatomy, and the need for advanced suturing skills. The introduction of RAP in the early 2000s marked a pivotal advancement, combining minimally invasive benefits with enhanced surgical precision. Redo RAP has consistently demonstrated higher success rates, fewer complications, and faster recovery compared with endopyelotomy and conventional laparoscopic techniques [9, 10].

The historical progression from open redo pyeloplasty to endopyelotomy, laparoscopic surgery, and now robotic‐assisted approaches reflects continuous efforts to balance surgical efficacy with patient safety, minimal invasiveness, and improved recovery. Moreover, progress in this field continues. New robotic platforms are under development, and innovations aim to make treatments even less invasive. With technology continuing to evolve at a rapid pace, further innovations in this area are just around the corner.

## 3 Cause of Recurrent Obstruction

### 3.1 Scar Tissue and Fibrosis

Scar tissue formation and fibrosis following initial surgical intervention for UPJO are among the most common causes of recurrence.

Elsawy identified scar tissue and fibrosis as the leading causes of recurrent UPJO after initial surgical correction. Excessive postoperative inflammation, infection, urinary extravasation, or suboptimal healing at the anastomotic site often results in fibrosis, which distorts anatomical landmarks and complicates subsequent surgeries [17].

The use and duration of ureteral stenting and intraperitoneal drainage during the initial procedure may influence the development of these postoperative complications.

Zhang underscored the importance of meticulous dissection through dense adhesions, noting that fibrosis significantly increases operative complexity and duration [18].

### 3.2 Crossing Vessels and Anatomical Factors

Crossing vessels—particularly aberrant lower‐pole renal arteries—are a significant anatomical cause of recurrent UPJO.

Hemal noted that these vessels can externally compress the ureteropelvic junction, leading to persistent or recurrent obstruction if not correctly identified and managed during the initial surgery [10].

Nayyar emphasized the necessity of detailed preoperative imaging, typically using computed tomography angiography or magnetic resonance angiography, to detect these vessels and guide intraoperative decision‐making [19]. In addition, Dy highlighted that anatomical anomalies such as horseshoe kidneys, duplicated collecting systems, malrotated kidneys, or unusual ureteral positioning further complicate surgical repair and require specialized surgical strategies [3].

Given these complexities, thorough preoperative assessment—including high‐resolution imaging, careful review of previous surgical records, and precise identification of recurrence causes—is paramount for optimizing surgical planning and enhancing outcomes in redo pyeloplasty.

### 3.3 Technical Failures During Initial Surgery

Technical inadequacies during primary pyeloplasty significantly increase recurrence risk. Sforza et al. identified common errors such as insufficient excision of the obstructed ureteral segment, inadequate mobilization of the renal pelvis and ureter, improper spatulation of the ureteral ends, suboptimal suturing techniques, and anastomotic tension resulting from either limited ureteral mobilization or excessive pelvic tissue reduction [20, 21].

Sundaram underscored the importance of meticulous technique, including precise dissection, adequate mobilization, proper alignment, and a tension‐free anastomosis, to minimize recurrence risks [6].

Multiple studies have consistently indicated that technical insufficiencies and suboptimal surgical execution during the initial procedure are associated with an increased risk of recurrence. Accordingly, it may be asserted that thorough and atraumatic dissection—when adhesions and fibrosis are minimal—combined with advanced suturing proficiency, is essential to minimize the likelihood of future obstruction.

## 4 Traditional Procedures for Recurrent Obstruction

Redo pyeloplasty for recurrent UPJO may be performed using open surgery, conventional laparoscopy, robotic‐assisted techniques, or endourological procedures. Each approach has distinct advantages and limitations regarding technical feasibility, surgical outcomes, complication rates, and postoperative recovery, making careful patient selection critical (Table 1). In open and laparoscopic redo pyeloplasty, the transperitoneal approach is generally preferred because it has a broader operative field and better exposure of the renal pelvis and proximal ureter. Retroperitoneal access may be considered in patients who previously underwent peritoneal surgery or to minimize bowel manipulation, although it offers limited workspace.

**TABLE 1:** Comparison of outcomes between robot‐assisted, laparoscopic, open redo pyeloplasty, and endopyelotomy for recurrent UPJO.

	Robot‐assisted	Laparoscopic	Open	Endopyelotomy
Success rate (%)	86–100	60–100	79–100	60–87
Operative time	Moderate to long	Long	Moderate	Short
Technical difficulty	Moderate	High	Moderate to high	Low
Length of hospital stay	Short (1–3 days)	Short to moderate (2–4 days)	Long (4–7 days)	Short (1–2 days)
Postoperative recovery	Rapid	Moderate	Slow	Rapid
Complication rate (%)	Low to moderate (5%–20%)	Moderate (10%–20%)	Moderate to high (20%)	Moderate (10%–15%)
Bleeding risk	Low	Low	Moderate	Moderate to high
Risk of recurrent obstruction	Low (5%–10%)	Moderate (10%–15%)	Low (5%–10%)	High (25%–40%)
Suitability for complex cases	Excellent	Good	Excellent	Limited

Abbreviation: UPJO, ureteropelvic junction obstruction.

### 4.1 Open Redo Pyeloplasty

Historically, open redo pyeloplasty has been considered the gold standard for treating recurrent UPJO due to its versatility, allowing surgeons to effectively manage complex anatomical distortions and significant fibrosis. Success rates ranged between 79% and 100%; however, the open approach is associated with considerable morbidity, including increased postoperative pain, longer hospital stays, prolonged convalescence periods, and poorer cosmetic outcomes [5, 22, 23]. Consequently, minimally invasive techniques are increasingly preferred in redo surgeries to minimize these drawbacks.

### 4.2 Endourological Approaches (Redo Endopyelotomy)

Redo endopyelotomy, performed via either antegrade percutaneous or retrograde ureteroscopic approaches, is the least invasive alternative for treating recurrent UPJO. It offers shorter operative times and faster recovery; however, its success rates—typically 60%–87%—are generally lower than those of open or laparoscopic redo pyeloplasty [1]. Moreover, endopyelotomy is significantly less effective in the presence of fibrosis, long strictures, or crossing vessels and carries risks such as vascular injury and significant bleeding, limiting its universal applicability [13].

### 4.3 Laparoscopic Redo Pyeloplasty

Laparoscopic redo pyeloplasty provides a minimally invasive alternative, significantly reducing postoperative pain, hospital stay, and recovery time compared with open surgery [5, 15]. However, this approach remains technically demanding in redo cases owing to extensive fibrosis, altered anatomy, and disrupted tissue planes. Limitations include difficulties in precise intracorporeal suturing, prolonged operative times, and a steep learning curve. Reported success rates range from 80% to 100%; however, outcomes are highly dependent on surgeon experience and patient selection [6] (Table 2).

**TABLE 2:** The study characteristics and outcomes for the redo LP data from articles.

Author	Year	Cases	Age (y)	Operative time (min)	Success rate (%)	Complication rate (%)	Hospital stay (days)
Piaggio [15]	2007	6	7.5	290	80	0	2.5
Powell [24]	2015	5	3.8	190	100	0	1.2
Nishi [7]	2015	9	27.5	241	100	22.2	6.1
Abraham [25]	2015	15	16.03	191.25	93.3	—	3.2
Abdel‐Karim [5]	2016	24	13.2	211.4	91.6	20.8	4
Moscardi [26]	2017	11	6.9	203.3	90.9	0	4.7
Hammady [27]	2017	32	29	133	90.6	21.9	2.7
Ambani [28]	2017	10	39.6	246.8	60.0	10	—
Chandrasekharam [29]	2020	9	3.9	109.3	100	22	2
Al‐Hazmi [30]	2020	22	1.9	200	91	0	3

Abbreviation: LP, laparoscopic pyeloplasty.

## 5 Redo Robotic‐Assisted Laparoscopic Pyeloplasty (Table 3)

### 5.1 Indications and Contraindications for Redo RAP

Redo RAP is particularly suitable for patients with localized ureteropelvic obstruction, preserved renal function, and absence of severe anatomical distortion. Ideal candidates include those with focal stricture segments who previously failed open or endoscopic procedures and have adequate renal pelvis volume. Conversely, redo RAP may be suboptimal in cases of extensive periureteral fibrosis, severely atrophic kidneys, anomalous vascular configurations, or long obliterative strictures where native ureteral reconstruction is not feasible. In such settings, alternative robotic techniques may be more appropriate.

**TABLE 3:** The study characteristics and outcome for the redo RAP data from articles.

Author	Year	Cases	Age (y)	Operative time (min)	Success rate (%)	Complication rate (%)	Hospital stay (days)
Hemal [10]	2008	9	17.9	106	100	0	3.4
Mufarrij [31]	2008	23	40	215.9	91.3	0	2.1
Sivaraman [32]	2011	21	36.0	190.4	95.2	14.3	1.7
Lindgren [33]	2012	16	6.5	303	90〜95	16.7	1.6
Baek [34]	2018	10	8.2	187.7	100	20.0	1.2
Jacobson [35]	2019	36	3.7	285.0	91.2	16.7	1.0
Zhang [18]	2020	32	24	151	96.9	9.4	4.0
Lee [36]	2020	28	52	188	85.7	0	1.0
Mittal [9]	2021	30	56.3	278	95.2	16.7	2.0
Dirie [37]	2021	13	27.5	170.5	75	23.1	6.6

Abbreviation: RAP, robot‐assisted pyeloplasty.

### 5.2 Surgical Techniques

Redo pyeloplasty benefits significantly from robotic assistance, which provides enhanced visualization, improved instrument control, and precise intracorporeal suturing. However, these procedures remain technically demanding, as surgeons must navigate dense fibrosis, disrupt anatomical landmarks, and potentially compromised vascular structures from prior surgeries.

The Anderson–Hynes dismembered pyeloplasty remains the most widely adopted technique in redo RAP and is typically performed via a transperitoneal approach using a standard three‐arm port configuration. Port placement generally involves an umbilical camera port flanked by two robotic working ports positioned along or just lateral to the midline. Assistant ports may be added based on surgeon preference and case complexity [9].

After gaining access to the retroperitoneal space, the ureter is carefully dissected beyond the fibrotic segment, and the renal pelvis is mobilized to allow a tension‐free anastomosis. The ureter is then spatulated and anastomosed to the dependent portion of the renal pelvis using 4‐0 or 5‐0 absorbable sutures, placed in a running or interrupted fashion. Antegrade placement of a double‐J stent is routinely performed, and surgical drains are selectively placed based on intraoperative findings.

In primary RAP for UPJO, instruments such as Black Diamond micro forceps and Potts scissors are commonly used for delicate manipulation and precise incision of the renal pelvis and ureter. However, in redo procedures involving dense fibrosis and scarring, this configuration may limit instrument control and increase the risk of inadvertent tissue trauma. Accordingly, the use of Maryland bipolar forceps for tissue handling and monopolar curved scissors for incision has shown improved operative control and reduced risk of crush injury.

Alternative reconstructive strategies, such as Y‐V plasty, may be considered in select cases—particularly when high ureteral insertion precludes dismembered repair. However, dismembered techniques remain preferred in most redo cases because of their superior adaptability in fibrotic fields.

Intraoperative evaluation of vascular anatomy is essential. While many patients do not exhibit crossing vessels due to prior correction, newly developed or persistent vascular compression may require posterior transposition of the ureter. Intraoperative assessment of ureteral perfusion can be challenging, particularly in redo cases. The use of indocyanine green (ICG) fluorescence imaging has been reported as a promising adjunct to identify well‐perfused ureteral segments suitable for anastomosis [38]. It is more commonly used in bowel segment selection during RA‐ileal ureter replacement (IUR); nevertheless, several centers have reported its utility in redo pyeloplasty to help minimize ischemia‐related anastomotic complications [39].

Overall, the robotic platform offers significant advantages in redo pyeloplasty by enabling meticulous dissection, precise suturing, and preservation of critical structures—all of which contribute to successful outcomes, even in highly complex cases.

### 5.3 Success Rates

Redo RAP has consistently demonstrated high success rates—ranging from 87% to 95%—with effective symptom relief and improved renal drainage. These outcomes establish redo RAP as a reliable option for patients with recurrent UPJO, including anatomically complex or previously operated cases.

Atug reported comparable success rates, blood loss, and hospital stays between primary and redo procedures. Redo surgeries required approximately 60 min longer owing to fibrosis and adhesions; however, the findings support robotic surgery safety and precision [40].

Lindgren reported that redo RAP achieved high success rates of approximately 90%–95% in pediatric patients previously treated for UPJO. Success was defined by both clinical improvement (resolution of pain or infections) and radiological evidence of unobstructed drainage on postoperative MAG3 renogram. Despite anatomical complexities and previous interventions, the study demonstrated consistently favorable outcomes with the robotic approach, emphasizing the importance of precise technique facilitated by robotic assistance [33].

Similarly, comparative analyses such as the study by Davis reported success rates of approximately 87%, noting that previous operative history significantly affects outcomes. Patients with extensive perirenal fibrosis or multiple previous interventions tend to exhibit lower success rates, underscoring fibrosis as a significant prognostic factor [41].

Passerotti described a redo RAP technique using a holding stitch on the renal pelvis to facilitate suturing and stenting, achieving effective symptom relief, no complications, and short hospital stays averaging 2 days [16]. Overall, the success rates of redo RAP compare favorably with conventional laparoscopic or open redo procedures.

In a systematic review and meta‐analysis by Autorino, RAP (primary and redo) demonstrated superior or comparable outcomes to traditional laparoscopic approaches, with success rates frequently exceeding 90% across multiple studies. These findings suggest that enhanced visualization, precision dissection, and meticulous anastomotic suturing, achievable with robotic instruments, substantially contribute to the high success rates observed [2].

Several factors directly influence these outcomes, including the surgical learning curve, surgeon experience, careful patient selection, and the degree of fibrosis encountered during the procedure. As reported in multiple studies, centers with higher volumes of robotic surgeries and redo cases tend to report better outcomes. Therefore, complex redo procedures should be performed in specialized centers to maximize the probability of success.

### 5.4 Complication Rates

Complication rates following redo RAP are generally considered acceptable and, in some studies, even lower than those associated with conventional laparoscopic or open surgical techniques. However, given the technical complexity of redo cases and variability in patient‐specific factors, a thorough understanding of potential complications is essential for effective patient counseling and preoperative planning.

Lindgren reported a perioperative complication rate of approximately 16.7% in a pediatric redo series, consisting mainly of minor events such as transient hematuria, urinary leakage, or infections—most of which resolved with conservative management or minimal intervention. Notably, major complications—defined as Clavien‐Dindo Grade III or higher—were uncommon, underscoring the overall safety profile of redo RAP [33].

Similarly, Davis reported a complication rate of approximately 21.8% among pediatric patients undergoing redo RAP. Most complications were manageable postoperative events, such as urinary tract infections, transient urinary leaks, ileus, and minor bleeding. Severe intraoperative complications—such as significant vascular injuries or damage to adjacent organs—were notably rare, highlighting the precision and safety advantages of robotic surgery [41].

Several factors may significantly influence complication rates. The extent of fibrosis and scarring from previous surgeries is a major determinant, as extensive adhesions can lead to prolonged operative time, increased intraoperative blood loss, and inadvertent tissue damage. Furthermore, anatomical anomalies, the presence of crossing vessels, and distorted anatomical landmarks associated with recurrent UPJO can further complicate the procedure and elevate complication risks.

Despite these inherent challenges, robot‐assisted surgery—with its enhanced visualization and precision—substantially mitigates complications during difficult dissections and reconstructions.

In a comparative analysis, Autorino found that robotic surgery was associated with fewer complications than conventional laparoscopic redo pyeloplasty, likely due to improved dexterity, tremor filtration, and high‐definition three‐dimensional imaging, allowing meticulous dissection and precise anastomosis even in highly fibrotic tissues [2]. Short‐term postoperative complications, such as minor leaks or transient hematuria, were typically managed conservatively and resolved without long‐term adverse effects. Long‐term complications—including recurrent obstruction or stricture necessitating further surgical intervention—were less common but were associated with significant fibrosis or technical challenges during the robotic redo procedure.

## 6 Alternative Techniques

Redo pyeloplasty presents significant technical challenges due to a range of factors. When conventional redo procedures are deemed infeasible or unlikely to yield durable success—such as in patients with multiple prior interventions, extensive ureteral strictures, or anomalous ureteropelvic anatomy—alternative reconstructive strategies should be considered. These include ureterocalicostomy, buccal mucosal graft ureteroplasty, IUR, and renal autotransplantation. Each technique offers distinct advantages and limitations, and the selection of the appropriate procedure requires careful assessment of individual anatomical and functional parameters. A detailed understanding of the indications, technical challenges, perioperative outcomes, and long‐term success rates of these approaches is essential for optimizing management in complex cases of recurrent UPJO.

### 6.1 Robot‐Assisted Ureterocalicostomy (Table 4)

Robot‐assisted ureterocalicostomy (RAUC) is indicated when the renal pelvis is intrarenal, severely scarred, or too small to permit tension‐free anastomosis. It is also considered in patients with high ureteral insertion or those who previously underwent multiple failed reconstructions. It has traditionally been considered a final option for renal preservation, serving as an alternative to nephrectomy [47].

**TABLE 4:** Robot‐assisted ureterocalicostomy as a redo procedure for failed pyeloplasty.

Author	Year	Cases	Age (y)	Operative time (min)	Success rate (%)	Complication rate (%)	Hospital stay (days)
Casale [42]	2008	6 (9)	6.5	168	100	—	0.9
Chhabra [43]	2016	5	33.7	172	83.3	60	6.5
Ramanitharan [44]	2020	6	33.7	178	100	33	6.1
Mittal [45]	2022	24	5.1	272	92	13	2
Xu [46]	2024	4 (6)	47	248.5	66.7	16.7	1

Casale was the first to describe RAUC in pediatric patients, adapting the procedure from an established open surgical technique [42]. The authors retrospectively reviewed nine pediatric patients who underwent RAUC for recurrent or intrarenal ureteral obstruction. Two procedures included concurrent ureteroscopic stone removal. The renal hilum was fixed to enable rapid vascular control; however, no hilum clamping was required in any case. At 12 months postoperatively, a diuretic renogram confirmed unobstructed drainage in all patients.

Chhabra also reported favorable outcomes in redo RAUC. In a series evaluating redo RAUC, the operative time averaged approximately 170 min, with minimal blood loss (approximately 100 mL) and a low conversion rate to open surgery. Most postoperative complications were minor; however, some patients required reintervention, such as balloon dilation or re‐stenting, which were categorized as Clavien–Dindo grade IIIb events. Functional success rates were encouraging, with most patients demonstrating good urinary drainage at short‐ to mid‐term follow‐up [43].

### 6.2 Robot‐Assisted Buccal Mucosal Graft Pyeloplasty (RA‐BMG) (Table 5)

RA‐BMG is typically chosen in cases of long‐segment ureteral strictures (> 3–4 cm), dense periureteral fibrosis, or complete obliteration of the ureteral lumen. It is also effective when local tissue is inadequate for a tension‐free primary anastomosis. Multiple studies have demonstrated the feasibility and efficacy of BMG in redo pyeloplasty.

**TABLE 5:** Robot‐assisted laparoscopic pyeloplasty incorporating a buccal mucosa graft for ureteral reconstruction.

Author	Year	Cases (redo)	Age (y)	Operative time (min)	Success rate (%)	EBL (mL)	Complication rate (%)	Hospital stay (days)
Lee [48]	2017	12 (8)	56	217	83.3	100	16.7	1
Lee [49]	2018	19 (10)	49	200	89	95	20	2
Chen [50]	2024	1	37	240	100	100	0	4
Chao [51]	2024	163 (77)	55	224–236	88.1–97.1	50	4.8	1
Grosso [52]	2025	10	58	165	90	175	20	5

Abbreviation: EBL, estimated blood loss.

Zhao reported a multi‐institutional series involving 19 patients undergoing RA‐BMG, more than half of whom had failed previous ureteral surgeries [49]. With a median stricture length of 4 cm, the procedure achieved a 90% success rate at a median follow‐up of 26 months.

Similarly, Lee evaluated 12 patients, including eight with prior failed reconstructions, achieving clinical and radiographic success in 83.3% of cases [48]. These findings support RA‐BMG as a safe and effective option for reconstructing the proximal and mid‐ureter.

The procedure is typically performed either as an onlay graft over an incised stricture or as an augmented anastomotic repair when the ureteral lumen is obliterated. In both cases, a pedicled omental flap is often used to provide vascular support to the graft. Importantly, the robotic platform facilitates fine dissection, minimizes tissue trauma, and enhances anastomotic precision—advantages particularly valuable in reoperative fields.

In a comparative study conducted across two high‐volume centers, Grosso et al. evaluated 26 patients who underwent BMG ureteroplasty: 16 using an open approach and 10 using a robotic approach. Patients in the RA‐BMG group presented with more complex characteristics, including significantly longer strictures (median: 26 mm vs. 17 mm) and a higher rate of prior reconstructive surgeries (80% vs. 37.5%). Despite these challenges, both groups achieved similarly high success rates (90.0% in the RA‐BMG group vs. 93.7% in the open‐BMG group). A notable advantage of the RA‐BMG group was significantly lower intraoperative blood loss (median: 175 mL vs. 300 mL), highlighting the potential benefits of a minimally invasive technique even in technically complex cases [52].

### 6.3 Robot‐Assisted Ileal Ureter Replacement (RA‐IUR) (Table 6)

RA‐IUR is reserved for complex cases with extensive ureteral loss, multiple failed prior reconstructions, or when neither ureterocalicostomy nor graft‐based repair is feasible. It is particularly useful when long‐segment replacement is required without compromising renal function. The procedure involves substituting the strictured ureteral segment with a detubularized or isoperistaltic segment of ileum, fully mobilized and anastomosed to both the renal pelvis and urinary bladder. Unlike open surgery, the robotic platform enables a minimally invasive, fully intracorporeal approach with enhanced dexterity, magnified visualization, and ergonomic suturing—key advantages when managing adhesions and performing complex anastomoses.

**TABLE 6:** Robot‐assisted ileal ureter replacement as a reconstructive option for ureteral stricture or recurrent ureteropelvic junction obstruction.

Author	Year	Cases	Age (y)	Operative time (min)	Success rate (%)	Complication rate (%)	Hospital stay (days)
Brandao [53]	2014	1	50	420	100	0	4
Chopra [54]	2016	3	50.3	470	100	33.3	7.7
Ubrig [55]	2018	7	61.1	328	100	0	13.9
Grosso [52]	2021	3	42.3	270	100	0	7
Yang [56]	2023	15	44	261.8	100	1/15	10.5

Several recent studies have demonstrated the feasibility and safety of RA‐IUR in redo UPJO cases. Ubrig reported a series of seven patients, including one with a solitary kidney, who underwent totally intracorporeal RA‐IUR. The median operative time was approximately 328 min, with blood loss (50–200 mL) and no conversions to open surgery. Notably, all patients maintained or improved renal function during follow‐up, with no major complications or need for reintervention [55].

Similarly, Chopra described a three‐patient series using a four‐arm robotic approach. One patient developed a bowel volvulus due to mesenteric herniation, necessitating bowel resection, while the remaining cases had favorable outcomes—supporting the technical viability of this method [54].

Yang expanded on this evidence in a larger cohort that included unilateral and bilateral RA‐IUR, some with concurrent ileocystoplasty [56]. At a median follow‐up of 14 months, they reported a 100% subjective success rate and an 86.7% functional success rate, emphasizing the procedure's reproducibility across various presentations [56].

Despite these successes, RA‐IUR is not without technical demands. Key considerations include ensuring an isoperistaltic configuration of the bowel segment, meticulous preservation of vascular supply, and avoiding partial substitutions, which have been associated with anastomotic stricture. Intraoperative use of ICG fluorescence imaging has proven beneficial in assessing bowel perfusion and reducing ischemia‐related complications.

RA‐IUR offers a robust reconstructive solution for recurrent UPJO when conventional interventions fail. With careful patient selection, adherence to refined surgical principles, and increasing experience among robotic surgeons, RA‐IUR is poised to become a cornerstone in the management of complex ureteral pathology.

### 6.4 Robot‐Assisted Kidney Autotransplantation (Table 7)

Robot‐assisted kidney autotransplantation (RA‐KAT) is a highly specialized salvage procedure that may be considered when all conventional reconstructive options—such as dismembered pyeloplasty, ureterocalicostomy, or ileal ureter replacement—are deemed unsuitable. Typical indications include long‐segment ureteral strictures exceeding 10–12 cm, devascularized ureters due to iatrogenic injury, or recurrent failures following multiple prior reconstructions, particularly in patients with a solitary kidney. It is not a standard treatment for UPJO; nonetheless, it may be applied in exceptionally complex cases of recurrent UPJO where no reconstructible ureteral segment remains and other options are not feasible. The first report of a fully intracorporeal RA‐KAT was published by Gordon [57], and the patient was a 56‐year‐old man with a 12‐cm devitalized ureter following ureteroscopy (not a classic case of UPJO). RA‐KAT has also been applied to complex and recurrent UPJO cases in which the ureter is not amenable to reconstruction using native tissue or alternative techniques. In such scenarios, it provides a kidney‐sparing alternative when the proximal ureter or renal pelvis remains functional. When RA‐KAT is used for UPJO, urinary tract reconstruction typically involves direct anastomosis of the renal pelvis—or, when feasible, the remaining ureter—to the bladder. The kidney is reimplanted into the iliac fossa, with vascular anastomoses performed to the external iliac vessels. Cold perfusion with preservation solution is used during intracorporeal or extracorporeal phases to minimize ischemic injury.

**TABLE 7:** Robot‐assisted renal autotransplantation for ureteral stricture.

Author	Year	Cases (U‐structure)	Age (y)/sex	Stricture length (cm)	Operative time (min)	Complication rate (%)	WIT/TIT (min)	Hospital stay (days)
Gordon [57]	2014	1	58/M	12	425	0	2.3/95.5	1
Lee [58]	2015	1	38/F	3.5	390	—	4/52	5
Araki [59]	2017	1	38/F	2.7	507 (console)	0	4/254	11
Decaestecker [60]	2018	7 (5)	20–47 M3/F4	3–12.9	370	42.9	2/180	5
Praet [61]	2020	1	58/F	—	345	—	4/59	—
Breda [62]	2022	29 (19)	28–55 M11/F18	9.2 (5.4–13.7)	350	31	3/111	5

Abbreviations: TIT, total ischemic time; U‐stricture, ureteral stricture; WIT, warm ischemic time.

Decaestecker presented a larger European case series involving seven patients, primarily with recurrent ureteral strictures. Of these, six underwent extracorporeal RA‐KAT and one a fully intracorporeal approach. The median operative time was 370 min, and renal function was preserved in all cases. Only one major complication—wound dehiscence—was reported, and all patients were stent‐ and symptom‐free at 3 months [60].

More recently, the European Robotic Urology Section (ERUS) published the largest comparative study of extracorporeal versus intracorporeal RA‐KAT (29 patients). Both approaches demonstrated acceptable complication rates (~13.8% for Clavien‐Dindo grade > II); however, extracorporeal RA‐KAT showed superior renal function preservation at 90 days, particularly in patients requiring complex vascular reconstruction. However, renal function outcomes at 1 year were comparable between the two groups [62]. These findings highlight RA‐KAT as a feasible and safe minimally invasive alternative for selected patients with recurrent or complex UPJO. Compared with open surgery, RA‐KAT offers several advantages, including smaller incisions, reduced blood loss, shorter hospital stays, and excellent functional outcomes. However, the RA‐KAT application is limited by its technical complexity, requiring expertise in robotic surgery and renal transplantation. As robotic platforms advance and surgical teams gain experience, the role of RA‐KAT is expected to expand. At present, it remains a valuable kidney‐sparing salvage option for recurrent UPJO when conventional reconstructions have failed.

### 6.5 Single‐Port Surgery

Single‐port robot‐assisted pyeloplasty (SP‐RAP) is emerging as a promising alternative to multiport (MP)‐RAP with benefits such as reduced invasiveness and improved cosmetic outcomes. SP‐RAP has been increasingly applied in selected cases of recurrent UPJO, including salvage procedures; however, it is not strictly an alternative to conventional redo RAP. A recent comparative analysis of perioperative and postoperative outcomes between SP‐ and MP‐RAP revealed that, although SP‐RAP is associated with a longer median operative time, it achieves comparable clinical outcomes and maintains an acceptable safety profile [63].

The SP robotic platform enables salvage pyeloplasty via either a transperitoneal or retroperitoneal approach. Among these, the supine anterior retroperitoneal access (SARA)—which involves an incision near McBurney's point—has gained popularity among SP surgeons because of its simplified access to the renal hilum, reduced risk of peritoneal adhesions from prior surgeries, and favorable cosmetic results.

Clinical reports on the use of the da Vinci SP system (Intuitive Surgical Inc., Sunnyvale, CA, USA) remain limited; however, their number is expected to grow as institutional adoption increases. SP‐RAP may be challenging in cases involving extensive adhesions, multi‐quadrant dissection, or complex vascular reconstruction.

Previously considered unsuitable for pediatric patients, SP‐RAP has now been shown to be feasible in selected older children and adolescents with adequate body habitus. The main limitations in smaller children relate to restricted working space and the use of instruments designed for adult anatomy. However, with advancing technology and growing surgical experience, broader pediatric applications may become feasible.

Single‐port robotic ureterocalycostomy, a more recent development, has been particularly notable in redo cases. Studies have shown success rates of approximately 67% in redo cases, indicating feasibility and safety even in patients with complicated surgical histories.

While the current adoption of SP platforms is still limited, accumulating evidence supports its feasibility and safety in select redo cases. Careful patient selection, appropriate approach planning, and technical familiarity are essential for optimal outcomes.

## 7 Limitations

Despite the promising outcomes associated with redo RAP and its advanced adaptations—including RA‐BMG, RA‐IUR, RA‐KAT, and SP platforms—several limitations remain.

First, the current body of evidence primarily consists of retrospective, single‐institution case series with limited sample sizes. The absence of randomized controlled trials or multicenter prospective studies makes it difficult to generalize findings or establish robust clinical guidelines. Furthermore, reported outcomes may be influenced by surgeon experience, institutional volume, and patient selection, which vary significantly across studies. Another key limitation is the lack of long‐term data on renal function and patient‐reported outcomes. Few studies have evaluated essential parameters such as progression to chronic kidney disease, blood pressure control, or quality of life beyond the early postoperative period. This gap is particularly concerning in pediatric patients, who require long‐term follow‐up to ensure renal function is preserved into adulthood [64]. Furthermore, the high cost and limited availability of robotic platforms remain significant barriers, particularly in resource‐limited settings or healthcare systems where robotic procedures are not reimbursed. Furthermore, advanced procedures such as RA‐IUR and RA‐KAT remain technically complex and are currently performed only in highly specialized centers, limiting broader adoption.

An additional limitation involves the constraints of current robotic platforms. At present, no robotic systems are specifically designed for pediatric patients, requiring surgeons to adapt adult‐sized instruments and platforms for use in much smaller anatomical spaces. This adaptation increases the technical complexity of pediatric redo RAP and may compromise surgical precision and safety. The absence of pediatric‐specific robotic systems highlights a significant technological gap. The development of miniaturized, child‐friendly surgical robots should be considered a future priority, particularly given that redo pyeloplasty is often required in children who previously underwent failed repairs at a young age.

To ensure continued progress, standardized protocols, pediatric‐specific innovations, and long‐term multicenter data are needed to validate outcomes and support broader adoption of robotic techniques for recurrent UPJO.

In conclusion, RAP has emerged as a highly effective and less invasive option for patients with recurrent UPJO, particularly in cases where prior interventions have failed or anatomical complexity poses significant surgical challenges. Beyond the standard redo pyeloplasty, the expanding application of advanced robotic techniques including RAUC, RA‐BMG, RA‐IUR, and RA‐KAT has created new possibilities for individualized, kidney‐preserving reconstruction. Despite their technical complexity, these procedures serve as effective salvage options in selected patients, particularly when performed by experienced surgeons at specialized centers.

Looking forward, robotic reconstructive surgery is poised to play an even greater role in managing complex upper urinary tract obstructions. To support this evolution, further efforts are needed to establish standardized surgical protocols, develop pediatric‐specific robotic platforms, and generate robust, long‐term, multicenter outcome data. As technology continues to advance and surgical expertise spreads, robotic techniques are increasingly positioned as the standard procedure for both routine and highly complex cases—offering patients a better balance between surgical precision, recovery, and long‐term function.

## Author Contributions

**Daisuke Ishii:** conceptualization, investigation, methodology, validation, writing – original draft. **Kohei Mori:** project administration, supervision, writing – review and editing. **Izuru Shiba:** conceptualization, investigation, supervision. **Yutaka Shiono:** conceptualization, investigation, supervision. **Kazumasa Matsumoto:** conceptualization, investigation, project administration, supervision, validation, writing – review and editing.

## Conflicts of Interest

The authors declare no conflicts of interest.
